# Graph-Calibrated Differential Privacy for Correlated IoT Sensing Streams

**DOI:** 10.3390/s26175386

**Published:** 2026-08-26

**Authors:** Zhengxuan Chen, Junming Chen

**Affiliations:** 1 College of Liberal Arts and Sciences, University of Connecticut, Storrs, CT 06269, USA; zhengxuan.chen@uconn.edu; 2 Faculty of Humanities and Arts, Macau University of Science and Technology, Macao, China

**Keywords:** differential privacy, IoT data analytics, correlation-aware privacy, graph-calibrated sensitivity, adaptive privacy budget allocation, correlated sensing streams, utility-preserving perturbation

## Abstract

Internet of Things (IoT) streams contain temporal, spatial, and cross-channel dependencies that can support inference about an attribute even after a formally private release. Standard differential privacy does not assume statistical independence; its guarantee is relative to a declared neighboring relation and may protect a narrower unit than the secret of practical interest. This paper proposes Graph-Calibrated Differential Privacy (GC-DP), which uses a lag-aware component graph to rank empirical exposure, construct conservative component scores, and allocate a release budget. The formal mechanism fixes a graph-expanded input adjacency independently of the protected data and calibrates each released coordinate to a certified global sensitivity bound. The graph, threshold, empirical-influence scores, and utility scores are obtained from public calibration information or through an explicitly composed private-calibration budget. For the latter case, the paper specifies a bounded-vector Laplace calibration mechanism and separates its budget from the release budget. Experiments on four public IoT datasets evaluate downstream utility, attribute-inference performance, ablations, and computational cost. All reported values are empirical measurements averaged over ten independent noise draws. Under the evaluated component-occurrence adjacency and public-calibration protocol, GC-DP achieves higher task utility and lower attribute-inference performance than the included uniform and partially adaptive baselines across the tested privacy budgets.

## 1. Introduction

Internet of Things (IoT) infrastructures continuously collect fine-grained sensing streams from smartphones, wearables, smart buildings, industrial equipment, and environmental networks. Differential privacy (DP) limits the change in an output distribution when the dataset changes according to a declared neighboring relation [1,2]. The definition does not assume that records are statistically independent. The practical difficulty in correlated sensing is instead that a component-level or time-point-level relation may be narrower than the user, event, or dependency neighborhood that an application intends to protect. Correlated observations can also retain predictive information about an attribute without violating the stated DP inequality.

This paper calls the latter phenomenon correlation-assisted inference. Indoor light and CO_2_ jointly indicate occupancy, accelerometer and gyroscope features jointly describe motion, and nearby motes observe similar environmental trends. Pufferfish makes the protected secrets, discriminative pairs, and data-generating assumptions explicit [3,4]; Blowfish specifies protected distinctions through a policy graph [5]; and temporal analyses show how repeated releases interact with temporal dependence [6,7]. These results motivate an IoT mechanism whose formal privacy unit is explicit and whose empirical calibration recognizes cross-channel and lagged structure.

Dependence-aware DP, Bayesian DP, correlated DP, and dependent-tuple analyses formalize related concerns under different models [8,9,10]. Workload mechanisms and streaming mechanisms solve different release problems: the matrix mechanism optimizes a fixed linear workload [11], CTS-DP publishes correlated time series [12], and CGM uses temporally correlated Gaussian noise under (ϵ,δ)-local DP [13]. GC-DP instead targets the central release of multichannel, window-level representations. These methods are not interchangeable baselines unless the privacy unit, trust model, value of δ, workload, and release schedule are matched.

GC-DP represents sensing components by a weighted calibration graph $\hat{G}=\left(V,E,W\right)$ and distinguishes it from the fixed policy graph $G_{0}$ that declares adjacency. Weighted degree ${\bar{r}}_{i}$ is an allocation surrogate, not a formal leakage bound. A certified sensitivity $B_{G_{0},k}$ is derived for every released coordinate $q_{k}$, and a graph-expanded empirical influence ${\hat{\xi }}_{k}$ may only increase the calibrated scale $s_{k}=\max\left\{B_{G_{0},k},{\hat{\xi }}_{k}\right\}$. Local budgets $\epsilon_{k}$ are computed from exposure, influence, and validation utility while satisfying $\sum_{k}\epsilon_{k}\leq \epsilon_{\mathrm{rel}}$. The release is ${\hat{y}}_{k}=\Pi_{k}\left(q_{k}\left(D\right)\right)+\mathrm{Laplace}\left(0,s_{k}/\epsilon_{k}\right)$.

The practical contribution is the integration of lag-aware dependency estimation, conservative sensitivity certification, and risk–utility allocation in one IoT release pipeline. Absolute lagged Pearson correlation was selected because it is bounded, sign-invariant, interpretable, and computable in $O(M^{2}\left(2L+1\right)N)$ time; thresholding and a degree cap produce an auditable sparse graph. Compression reduces redundant empirical scores but never reduces the certified bound. These choices are pragmatic rather than universally optimal; mutual information, partial correlation, or domain graphs may be substituted when their calibration and sensitivity are specified. When graph or utility statistics use protected records, GC-DP assigns budgets $\epsilon_{G}$, $\epsilon_{\tau }$, $\epsilon_{\mu }$, and $\epsilon_{\xi }$ to their release, with $\epsilon_{\mathrm{cal}}=\epsilon_{G}+\epsilon_{\tau }+\epsilon_{\mu }+\epsilon_{\xi }$ and total cost $\epsilon_{\mathrm{cal}}+\epsilon_{\mathrm{rel}}$.

The evaluation uses UCI-HAR, WISDM, Occupancy Detection, and Intel Lab Data. The experimental implementation follows Algorithms 1–4: it applies the fixed component-occurrence adjacency, includes the certified coordinatewise sensitivity floor, and then uses graph exposure, empirical influence, and validation utility to allocate the release budget. The tables report higher utility and lower attribute-inference AUC for GC-DP than for Lap-DP, Gau-DP, Ada-DP, and Risk-DP. These comparisons concern the matched central-release implementations evaluated here; mechanisms with different secret definitions, trust models, workloads, values of δ, or release schedules remain scope comparisons rather than numerical baselines.

The contributions are as follows: (1) a query-independent graph-expanded adjacency that states the protected component-level neighborhood; (2) a separation between certified global sensitivity and empirical graph influence; (3) an explicit public/private calibration boundary and composed privacy accounting; and (4) an empirical evaluation of utility, attribute inference, ablations, and computational cost on four public IoT sensing datasets.

The remainder of this paper is organized as follows. Section 2 reviews differential privacy for IoT data analytics, correlation-aware privacy, and graph-structured budget allocation. Section 3 introduces the notation, IoT stream model, differential privacy background, and graph-based privacy–utility objective. Section 4 presents the GC-DP framework, including correlation graph construction, graph-calibrated sensitivity estimation, adaptive budget allocation, and the complete private release mechanism. Section 5 reports the experimental settings, utility comparison, privacy leakage analysis, ablation study, and runtime evaluation. Section 6 concludes the paper.
**Algorithm 1** Data-Driven Correlation Graph Construction**Require:** Public or privately calibrated bounded data/score vector, *L*, λ, τ, degree cap *K*, smoothing coefficient α **Ensure:** Calibration graph $\hat{G}$, normalized adjacency matrix $\tilde{W}$, graph exposure vector $\bar{r}$ 1:Apply fixed bounded normalization parameters. 2:Obtain $S_{ij}$ from public normalized data or read the already released private score vector $\tilde{S}$; do not access protected calibration records again. 3:Form undirected candidates $E_{\tau }=\left\{\left\{i,j\right\}:S_{ij}\geq \tau ,\;i<j\right\}$ and sort them by decreasing $S_{ij}$ with a lexicographic tie break. 4:Initialize W=0. In sorted order, accept {i,j} only if deg(i)<K and deg(j)<K, then set $w_{ij}=w_{ji}=S_{ij}$. 5:Compute $\tilde{W}=D^{-1/2}WD^{-1/2}$; use zero inverse degree for isolated nodes. 6:Compute direct weighted degree $d_{m}=\sum_{j\neq m}w_{mj}$ for each node $v_{m}$. 7:Compute smoothed exposure score $r_{m}=\alpha d_{m}+\left(1-\alpha \right)\sum_{j\neq m}{\tilde{w}}_{mj}d_{j}$. 8:Normalize $r_{m}$ into ${\bar{r}}_{m}\in \left[0,1\right]$ for all *m*. 9:**return** $\hat{G}=\left(V,E,W\right)$, $\tilde{W}$, and $\bar{r}$

**Algorithm 2** Graph-Calibrated Sensitivity Estimation**Require:** Query *q*, fixed adjacency ${∼}_{G_{0}}$, certified bounds $\left\{B_{G_{0},k}\right\}$, calibrated graph $\hat{G}$, exposure $\bar{r}$, public/private calibration data, $\gamma ,\tau_{c},\kappa ,g_{\max}$ **Ensure:** Certified bounds $\left\{B_{G_{0},k}\right\}$, empirical scores $\left\{{\hat{\xi }}_{k}\right\}$, release numerators $\left\{s_{k}\right\}$ 1:**for** each released coordinate *k* **do** 2:   Verify analytically that $B_{G_{0},k}\geq \Delta_{G_{0},k}\left(q\right)$; otherwise use the clipped range $U_{k}-L_{k}$. 3:   On public calibration data, compute the bounded mask score using fixed a(k) and $C_{k}$; in private mode, read the released coordinate of $V_{\xi }$ without accessing protected records again. 4:   Inflate and cap the public empirical score, or project the private score, to obtain ${\hat{\xi }}_{k}$. 5:   Set $s_{k}=\max\left\{B_{G_{0},k},{\hat{\xi }}_{k}\right\}$. 6:**end for** 7:**return** ${\left\{B_{G_{0},k},{\hat{\xi }}_{k},s_{k}\right\}}_{k=1}^{d}$

**Algorithm 3** Utility-Guided Adaptive Privacy Budget Allocation**Require:** $\epsilon_{\mathrm{rel}}$, calibrated $\bar{r}$, $\left\{s_{k}\right\}$, $\left\{\mu_{k}\right\}$, $\epsilon_{\min}$, $\beta_{r},\beta_{g},\beta_{u}$ **Ensure:** $\epsilon ={\left[\epsilon_{1},\ldots ,\epsilon_{d}\right]}^{⊤}$ 1:Verify that $0<\epsilon_{\min}<\epsilon_{\mathrm{rel}}/d$. 2:**for** each output coordinate *k* **do** 3:   Compute ${\bar{r}}_{k}^{\left(q\right)}$ from a(k), then $p_{k}={\left({\bar{r}}_{k}^{\left(q\right)}+\eta \right)}^{\beta_{r}}{\left(s_{k}+\eta \right)}^{\beta_{g}}$ and $u_{k}={\left(\mu_{k}+\eta \right)}^{\beta_{u}}$. 4:   Set $h_{k}=u_{k}/\left(p_{k}+\eta \right)$. 5:**end for** 6:Set $\epsilon_{k}=\epsilon_{\min}+\left(\epsilon_{\mathrm{rel}}-d\epsilon_{\min}\right)h_{k}/\sum_{j=1}^{d}h_{j}$. 7:**return** ϵ

**Algorithm 4** Graph-Calibrated Differential Privacy Mechanism**Require:** Protected data D, query *q*, fixed adjacency ${∼}_{G_{0}}$, release budget $\epsilon_{\mathrm{rel}}$, fixed/public or $\epsilon_{\mathrm{cal}}$-DP parameter object θ **Ensure:** Privatized analytics output $\hat{y}$ 1:Verify $s_{k}\geq B_{G_{0},k}\geq \Delta_{G_{0},k}\left(q\right)$, $\epsilon_{k}>0$, and $\sum_{k}\epsilon_{k}\leq \epsilon_{\mathrm{rel}}$ for every possible θ. 2:**for** each released coordinate k=1,…,d **do** 3:   Set $y_{k}=\Pi_{k}\left(q_{k}\left(D\right)\right)$ and $b_{k}=s_{k}/\epsilon_{k}$. 4:   Sample $Z_{k}∼\mathrm{Laplace}\left(0,b_{k}\right)$ independently and release ${\hat{y}}_{k}=y_{k}+Z_{k}$. 5:**end for** 6:**return** $\hat{y}={\left[{\hat{y}}_{1},\ldots ,{\hat{y}}_{d}\right]}^{⊤}$

## 2. Related Work

### 2.1. Differential Privacy for IoT Sensing, Streams, and Private Learning

The Laplace and Gaussian mechanisms calibrate noise to global sensitivity under an explicitly chosen adjacency, and sequential composition accounts for repeated access [1,2]. Continual-observation mechanisms address repeated stream releases [14,15], while event-level and *w*-event privacy distinguish one event from a bounded temporal interval [16]. Local DP changes the trust model by perturbing at the source [17,18]. These distinctions matter for IoT evaluation: a central pure-ϵ release is not directly comparable with an (ϵ,δ) local mechanism solely by matching ϵ.

GC-DP concerns a central release of clipped window-level components. It does not replace user-level, device-level, continual-observation, or local privacy; those guarantees require their own adjacency and accounting. The experimental tables compare mechanisms implemented within the same central-release pipeline and with the same data partitions and release budgets. Broader mechanisms are compared by scope because their privacy units, trust models, workloads, or release schedules differ.

### 2.2. Correlation-Aware Privacy for Dependent Spatiotemporal Data

Standard DP does not require records to be independent; dependence becomes relevant when adjacency protects a smaller unit than the intended secret or when correlated observations provide auxiliary evidence for inference. Pufferfish makes secrets, discriminative pairs, and distribution families explicit [3], and the Markov Quilt mechanism instantiates it for Bayesian networks and Markov chains [4]. Blowfish uses a policy graph over possible data values [5]; that graph is conceptually different from GC-DP’s empirical graph over sensing components. Bayesian DP, correlated DP, and dependent DP quantify dependence under different probabilistic or sensitivity models [8,9,10].

GC-DP fixes the protected expansion relation before observing the protected release data and uses the learned component graph only for calibration. This separation avoids defining adjacency through an observed query displacement. Graph degree and attribute-inference scores remain empirical indicators; they do not modify or amplify the meaning of the formal DP inequality after release.

Other peer-reviewed dependence-aware studies cover feature selection, mobile crowdsensing, correlated trajectories, and correlated multi-attribute estimation [19,20,21,22]. Recent private time-series work also distinguishes user-level local shape extraction from stream-publication reuse of perturbations [23,24]. These task-specific assumptions reinforce the need to state scope before comparing numerical utility.

### 2.3. Graph-Structured Privacy Modeling and Adaptive Budget Allocation

The matrix mechanism chooses strategy queries for a specified workload of linear counting queries [11]; it is not a generic classifier-release baseline. Location and temporal methods use geometry, Markov transitions, or temporal accounting [6,7]. CTS-DP constructs correlated Laplace noise for time-series publication [12], whereas CGM injects correlated Gaussian noise under (ϵ,δ)-local DP using public aggregate autocorrelation [13]. A fair empirical comparison requires the same workload, protected unit, trust model, δ, and schedule.

GC-DP is distinguished by a lag-aware graph over heterogeneous components, certified coordinatewise sensitivity under a fixed expanded adjacency, and task-aware budget allocation. The graph form was chosen to support transparent inspection and periodic reuse, not because weighted degree has a formal equivalence to leakage or because correlation graphs are optimal for every IoT process.

## 3. Preliminaries

This section distinguishes the declared privacy unit, fixed policy adjacency, learned calibration graph, input-node index, and output-coordinate index. Table 1 summarizes the notation.

### 3.1. IoT Sensing Streams and Analytics Queries

Let $D=\{x_{1},\ldots ,x_{N}\}$ contain clipped multivariate observations. Before calibration, the controller fixes atomic occurrence-level units I⊆{1,…,N}×V and their domains. A graph node $v_{i}$ is a component type, whereas an atomic unit $\left(t,v_{i}\right)$ is that component’s occurrence in record or window *t*; the two are not interchangeable. The experiments protect a supplied feature-window coordinate for UCI-HAR, a sensor-axis contribution within one WISDM window, a time-stamped channel value for Occupancy Detection, and a mote–channel contribution within one Intel Lab window. The guarantee is component-occurrence privacy, not user-level or device-level privacy. User- or device-level protection requires grouping all corresponding occurrences into one adjacency and generally has a larger sensitivity.

The released query is $q:D\to R^{d}$, with coordinate $q_{k}$ distinguished from input-node index *i*. Aggregate queries and clipped feature representations are both covered. A deterministic projection $\Pi_{k}$ maps coordinate *k* to its fixed interval $\left[L_{k},U_{k}\right]$ before randomization.

Let $\Gamma_{G_{0}}\left(u\right)\subseteq I$ be a predeclared expansion set obtained from a public policy graph $G_{0}$ or a specification independent of the protected data, where $u=(t,v_{i})$ is one atomic occurrence. Datasets satisfy $D{∼}_{G_{0}}D^{\prime }$ when, for one *u*, they agree outside $\Gamma_{G_{0}}\left(u\right)$ and may differ arbitrarily within the fixed clipped domains inside that set. The conventional component-replacement relation is recovered when $\Gamma_{G_{0}}\left(u\right)=\left\{u\right\}$. The relation is defined on input occurrences and does not depend on the query, observed displacement, or learned sensitivity. Raw-channel, temporal-segment, and feature-level graphs are separate instantiations and are not mixed within one run. In the benchmark experiments, $G_{0}$ and its occurrence-level expansions are fixed from the public data specification before calibration; the learned weighted graph $\hat{G}$ is used only for sensitivity inflation and budget allocation.

### 3.2. Differential Privacy Under Correlated Observations

A randomized mechanism M is ϵ-DP under ${∼}_{G_{0}}$ if $\mathrm{Pr}\left[M\left(D\right)\in S\right]\leq e^{\epsilon }\mathrm{Pr}\left[M\left(D^{\prime }\right)\in S\right]$ for every $D{∼}_{G_{0}}D^{\prime }$ and measurable S. The guarantee limits distinguishability for exactly those pairs. It does not state that an attribute is unrecoverable from correlations, nor does it automatically extend component-record privacy to a whole user or device.

Correlation-assisted attribute inference is evaluated separately from the formal guarantee. A lower attack AUC or F1 indicates weaker performance for the evaluated adversary, not absence of privacy risk. This distinction also explains why graph-weighted degree can guide allocation without being presented as a theorem-level measure of leakage.

### 3.3. Correlation Graph and Privacy–Utility Objective

The calibration graph $\hat{G}=\left(V,E,W\right)$ has *M* nodes and bounded weights $w_{ij}\in \left[0,1\right]$. Its node representation is fixed for each experiment. The graph ranks empirical dependence for budget allocation; the independent policy graph $G_{0}$ defines adjacency. The two coincide only when $\hat{G}$ is constructed entirely from public information and explicitly adopted as policy before the protected release data are observed.

The normalized degree score ${\bar{r}}_{i}$ is treated as an interpretable exposure surrogate. It is neither equal to nor an upper bound on privacy loss, mutual information, reconstruction success, or attack AUC. The ablation and attribute-inference experiments evaluate its empirical contribution, while the certified sensitivity bound remains the basis of the formal guarantee.

GC-DP distributes a release budget so that $\sum_{k=1}^{d}\epsilon_{k}\leq \epsilon_{\mathrm{rel}}$. Exposure and certified sensitivity decrease a coordinate’s allocation weight, while bounded validation utility increases it. If any of these quantities is obtained from protected records, its private calibration is included in $\epsilon_{\mathrm{cal}}$ before the allocation is used.

## 4. Methodology

### 4.1. Data-Driven Correlation Graph Construction

GC-DP constructs a bounded calibration graph $\hat{G}=\left(V,E,W\right)$ over the node representation fixed for the task. The graph ranks synchronous and lagged empirical dependence for allocation; it does not itself define privacy loss. Calibration and protected release are separate stages, and every calibration quantity is either independent of the protected records or released under an explicit calibration budget.

Let the calibration matrix contain $N_{\mathrm{cal}}$ bounded observations of *M* fixed nodes. Public medians and interquartile ranges may be used for robust normalization. If these statistics are computed from protected records, their release is included in the parameter object θ and budgeted; computing them first and then treating them as constants is not sufficient. Fixed physical ranges or public training statistics are preferred because they make clipping and sensitivity auditable.

For nodes *i* and *j*, let $\rho_{ij}^{\left(l\right)}$ be the absolute Pearson correlation at lag *ℓ* after fixed bounded preprocessing. Set $\rho_{ij}^{\left(l\right)}=0$ when fewer than two aligned pairs remain or either aligned series has zero variance. The bounded score is $S_{ij}=\lambda \rho_{ij}^{\left(0\right)}+\left(1-\lambda \right)\max_{|l|\leq L}\rho_{ij}^{\left(l\right)}$. After calibration, $w_{ij}=S_{ij}$ when $S_{ij}\geq \tau$ and zero otherwise. Absolute lagged correlation was selected because it is bounded, sign-invariant, inexpensive, and directly inspectable. It does not distinguish direct from mediated dependence, so partial correlation, mutual information, or a domain graph may be preferable when supported by the application and privacy accounting.

Thresholding first forms the undirected candidate set $E_{\tau }=\left\{\left\{i,j\right\}:S_{ij}\geq \tau ,\;i<j\right\}$. Candidates are sorted by decreasing $S_{ij}$, with lexicographic node order used as a deterministic tie-breaker. Starting from an empty undirected graph, an edge {i,j} is accepted only when both current endpoint degrees are strictly smaller than *K*. This greedy symmetric rule guarantees deg(i)≤K for every node; it does not reinstate sub-threshold neighbors, and isolated nodes are permitted. The accepted edge receives $w_{ij}=w_{ji}=S_{ij}$. Then $\tilde{W}=D^{-1/2}WD^{-1/2}$, with inverse-degree entries defined as zero for isolated nodes. The construction limits weak or spurious links while preserving an auditable sparse structure; it is a deterministic sparsification heuristic, not an optimality claim.

The allocation surrogate uses $d_{i}=\sum_{j\neq i}w_{ij}$ and $r_{i}=\alpha d_{i}+\left(1-\alpha \right)\sum_{j\neq i}{\tilde{w}}_{ij}d_{j}$. If $\max_{i}r_{i}>\min_{i}r_{i}$, min–max normalization gives ${\bar{r}}_{i}=\left(r_{i}-\min_{j}r_{j}\right)/\left(\max_{j}r_{j}-\min_{j}r_{j}\right)$; otherwise, set every ${\bar{r}}_{i}=0$. It ranks direct and one-hop graph exposure. No formal equivalence between weighted degree and DP leakage is assumed; the attack evaluation tests only whether the surrogate improves resistance for the specified empirical adversary.

Algorithm 1 receives an already public or privately released score vector and applies only post-processing. It returns $\hat{G}$, $\tilde{W}$, and $\bar{r}$.

Direct computation over M(M−1)/2 pairs, 2L+1 lags, and $N_{\mathrm{cal}}$ aligned observations costs $O(M^{2}\left(2L+1\right)N_{\mathrm{cal}})$ time. Thresholding and per-node ranking cost $O(M^{2}\log M)$ time; the dense scores require $O(M^{2})$ memory beyond the input. FFT-based correlation can reduce the lag term for long streams, but the reported implementation uses direct correlation.

### 4.2. Public and Private Calibration

Collect all release-dependent parameters in $\theta =(W,\bar{r},\mu ,\hat{\xi },\tau ,\epsilon )$. In public-calibration mode, θ, clipping bounds, and tuning choices use only public information independent of the protected records. A historical or validation split is not automatically public; if it contains protected units, it must be included in the accounting.

In private-calibration mode, fixed preprocessing maps the P=M(M−1)/2 pairwise scores to a vector $S\left(D\right)\in {\left[0,1\right]}^{P}$. Define $\Delta_{1}S=\sup_{D{∼}_{G_{0}}D^{\prime }}{∥S\left(D\right)-S\left(D^{\prime }\right)∥}_{1}$. GC-DP releases ${\tilde{S}}_{j}=\Pi_{\left[0,1\right]}\left(S_{j}\left(D\right)+Z_{j}\right)$ with independent $Z_{j}∼\mathrm{Laplace}\left(0,\Delta_{1}S/\epsilon_{G}\right)$. The universal bound $\Delta_{1}S\leq P$ is valid because every coordinate is in [0,1]; a tighter value may be used only when proved for the stated score and adjacency. Thresholding, degree capping, normalization, compression, and exposure scoring are post-processing. For utility and empirical-influence vectors $V_{\mu }\left(D\right),V_{\xi }\left(D\right)\in {\left[0,1\right]}^{d}$, define $\Delta_{1}V_{a}=\sup_{D{∼}_{G_{0}}D^{\prime }}{∥V_{a}\left(D\right)-V_{a}\left(D^{\prime }\right)∥}_{1}$ for a∈{μ,ξ}. Independent coordinatewise Laplace noise with scales $\Delta_{1}V_{\mu }/\epsilon_{\mu }$ and $\Delta_{1}V_{\xi }/\epsilon_{\xi }$, followed by projection to ${\left[0,1\right]}^{d}$, releases these vectors; the universal bounds are $\Delta_{1}V_{a}\leq d$.

If protected validation utility U(D,t)∈[0,1] selects τ from a finite set T, the exponential mechanism samples *t* with probability proportional to $\exp\{\epsilon_{\tau }U\left(D,t\right)/\left(2\Delta U\right)\}$, where $\Delta U=\sup_{D{∼}_{G_{0}}D^{\prime }}\left|U\left(D,t\right)-U\left(D^{\prime },t\right)\right|\leq 1$. The calibration budget is $\epsilon_{\mathrm{cal}}=\epsilon_{G}+\epsilon_{\tau }+\epsilon_{\mu }+\epsilon_{\xi }$. Clipping and normalization parameters must remain public/fixed or be included in a separately specified DP parameter release; they cannot redefine adjacency. Private graph calibration can be noisy under the universal bound, which is an explicit utility limitation rather than an unaccounted guarantee.

### 4.3. Graph-Calibrated Sensitivity Estimation

Let $q\left(D\right)=(q_{1}\left(D\right),\ldots ,q_{d}\left(D\right))$. For the fixed relation ${∼}_{G_{0}}$, the true sensitivity of output coordinate *k* is

$$\Delta_{G_{0},k}\left(q\right)=\underset{D{∼}_{G_{0}}D^{\prime }}{\sup}\left|\Pi_{k}\left(q_{k}\left(D\right)\right)-\Pi_{k}\left(q_{k}\left(D^{\prime }\right)\right)\right|.$$

A certificate $B_{G_{0},k}$ must satisfy $B_{G_{0},k}\geq \Delta_{G_{0},k}\left(q\right)$. Counts, fixed-size means, and histograms use analytic bounds from the declared adjacency and ranges. For an arbitrary extractor clipped to $\left[L_{k},U_{k}\right]$, $B_{G_{0},k}=U_{k}-L_{k}$ is always valid, although potentially loose. The vector query obeys $\Delta_{G_{0}}^{\left(1\right)}q\leq \sum_{k=1}^{d}B_{G_{0},k}$; in general, the maximum coordinate bound is not an *ℓ*_1_ vector-sensitivity bound.

Bounded leave-one-node masking measures observed task influence, not global sensitivity. Fix a mapping $a:\left\{1,\ldots ,d\right\}\to 2^{V}$ from every output coordinate to its associated graph nodes; when d=M, $a\left(k\right)=\left\{v_{k}\right\}$. Let $\delta_{ik}=\left|\Pi_{k}\left(q_{k}\left(D_{\mathrm{cal}}\right)\right)-\Pi_{k}\left(q_{k}\left(M_{i}\left(D_{\mathrm{cal}}\right)\right)\right)\right|$, where $M_{i}$ applies the prespecified mask to node type $v_{i}$. For a fixed compressed set $C_{k}\subseteq V$, define

$${\hat{\xi }}_{k}=\min\;\left\{g_{\max},\;\kappa \max_{i\in a(k)}\;\left[\delta_{ik}+\gamma {\bar{r}}_{i}\sum_{j\in C_{k}}{\tilde{w}}_{ij}\delta_{jk}\right]\right\}.$$

The set $C_{k}$ retains one representative from a pair with normalized similarity at least $\tau_{c}$, choosing the representative with greater exposure and using a fixed node-index tie break. This formula is evaluated on public calibration data; with protected calibration data, Algorithm 2 consumes the already released private vector $V_{\xi }$ from Section 4.2 rather than remasking protected records. Inflation by κ≥1 and clipping at $g_{\max}$ stabilize this empirical score, but no finite κ converts a validation maximum into a worst-case certificate. Compression and clipping therefore affect only ${\hat{\xi }}_{k}$. The release uses

$$s_{k}=\max\left\{B_{G_{0},k},{\hat{\xi }}_{k}\right\},$$

so empirical estimation error cannot lower the certified bound. The analytic route is used whenever the query algebra and adjacency yield a closed-form global bound; bounded perturbation is used only to rank influence for allocation or conservatively increase $s_{k}$.

This separation explains the practical role of graph expansion and compression. Expansion directs more noise or less budget toward coordinates linked to influential nodes, whereas compression avoids repeatedly counting nearly collinear evidence in the empirical score. Neither operation is claimed to be optimal, and neither is allowed to reduce formal sensitivity.

**Proposition** **1.** *For every coordinate k, $s_{k}\geq B_{G_{0},k}\geq \Delta_{G_{0},k}\left(q\right)$. Thus, $s_{k}$ is a valid coordinatewise sensitivity bound. Graph compression, masking error, and the choices of γ, $\tau_{c}$, κ, and $g_{\max}$ may change utility and empirical attack behavior but cannot invalidate this inequality because the independently certified term remains at the maximum.*

### 4.4. Utility-Guided Adaptive Privacy Budget Allocation

The certified scale numerators $s_{k}$ protect the declared adjacency. GC-DP then allocates $\epsilon_{\mathrm{rel}}$ across coordinates to trade task utility against empirical graph exposure without changing those certificates.

Let $\epsilon_{k}>0$ be the budget for output coordinate *k*, with $\sum_{k=1}^{d}\epsilon_{k}\leq \epsilon_{\mathrm{rel}}$. The bounded utility score $\mu_{k}\in \left[0,1\right]$ is the normalized reduction in validation utility when the associated node or feature group is masked; aggregates use a bounded contribution score. The score and resulting allocation are privacy-dependent. They must be computed from public information or released within $\epsilon_{\mu }$. A non-private allocation computed from the protected release data is outside Theorem 1.

Using the fixed coordinate–node map a(k) from Section 4.3, define coordinate exposure by

$${\bar{r}}_{k}^{\left(q\right)}=\frac{1}{\left|a(k)\right|}\sum_{v_{i}\in a\left(k\right)}{\bar{r}}_{i}.$$

Let $p_{k}={\left({\bar{r}}_{k}^{\left(q\right)}+\eta \right)}^{\beta_{r}}{\left(s_{k}+\eta \right)}^{\beta_{g}}$, $u_{k}={\left(\mu_{k}+\eta \right)}^{\beta_{u}}$, and $h_{k}=u_{k}/\left(p_{k}+\eta \right)$. Larger $p_{k}$ reduces the allocated budget and increases noise; larger $u_{k}$ preserves task-critical coordinates. The exponents $\beta_{r}$, $\beta_{g}$, and $\beta_{u}$ control the three effects.

With $0<\epsilon_{\min}<\epsilon_{\mathrm{rel}}/d$, allocation is

$$\epsilon_{k}=\epsilon_{\min}+\left(\epsilon_{\mathrm{rel}}-d\epsilon_{\min}\right)\frac{h_{k}}{\sum_{j=1}^{d}h_{j}}.$$

The floor prevents numerical degeneracy and the normalization makes the total budget exactly $\epsilon_{\mathrm{rel}}$.

When $\beta_{r}=0$, graph exposure does not affect allocation, giving the utility-only Ada-DP reduction; when $\beta_{u}=0$, validation utility does not affect it, giving the exposure-only Risk-DP reduction. The rule is a transparent allocation heuristic rather than the solution of a universal optimality criterion.

Algorithm 3 is the post-processing of the fixed or privately calibrated object θ and requires O(d) time and memory.

**Proposition** **2.** *Algorithm 3 guarantees $\epsilon_{k}\geq \epsilon_{\min}$ and $\sum_{k=1}^{d}\epsilon_{k}=\epsilon_{\mathrm{rel}}$. This follows immediately from positivity of $h_{k}$ and summing the normalized fractions.*

### 4.5. Complete Graph-Calibrated Private Release Mechanism

The release stage accepts a fixed or privately calibrated parameter object θ; it does not recompute graph, threshold, empirical influence, utility, clipping, or allocation statistics from the unprotected release data. For k=1,…,d, it releases

$${\hat{y}}_{k}=\Pi_{k}\left(q_{k}\left(D\right)\right)+Z_{k},\;Z_{k}\overset{\mathrm{ind}}{∼}\mathrm{Laplace}\left(0,s_{k}/\epsilon_{k}\right),$$

where $s_{k}\geq \Delta_{G_{0},k}\left(q\right)$ and $\sum_{k}\epsilon_{k}\leq \epsilon_{\mathrm{rel}}$. The graph may increase $s_{k}$ or decrease $\epsilon_{k}$, but it cannot lower the certified bound.

**Theorem** **1.** *Fix ${∼}_{G_{0}}$ independently of the protected data. Suppose that for every feasible fixed calibration output θ, each nonconstant coordinate has $s_{k}\left(\theta \right)>0$, $s_{k}\left(\theta \right)\geq \Delta_{G_{0},k}\left(q\right)$, $\epsilon_{k}\left(\theta \right)>0$, and $\sum_{k}\epsilon_{k}\left(\theta \right)\leq \epsilon_{\mathrm{rel}}$; a coordinate with zero sensitivity is released deterministically. Then the release kernel $R_{\theta }$ defined by Algorithm 4 is $\epsilon_{\mathrm{rel}}$-DP under ${∼}_{G_{0}}$ for every such fixed θ. If an $\epsilon_{\mathrm{cal}}$-DP calibration kernel C produces θ from the same protected records under the same relation, the joint adaptive procedure $C\left(D;d\theta \right)R_{\theta }\left(D;d\hat{y}\right)$, and hence its released marginal, is $\left(\epsilon_{\mathrm{cal}}+\epsilon_{\mathrm{rel}}\right)$-DP.*

**Proof.**  For fixed θ and any $D{∼}_{G_{0}}D^{\prime }$, independence of the Laplace variables gives

$$\frac{p_{\theta }\left(\hat{y}|D\right)}{p_{\theta }\left(\hat{y}|D^{\prime }\right)}\leq \exp\;\left(\sum_{k=1}^{d}\frac{\epsilon_{k}\left|\Pi_{k}\left(q_{k}\left(D\right)\right)-\Pi_{k}\left(q_{k}\left(D^{\prime }\right)\right)\right|}{s_{k}}\right)\leq \exp\;\left(\sum_{k=1}^{d}\epsilon_{k}\right)\leq e^{\epsilon_{\mathrm{rel}}}.$$

Integration gives the same inequality for measurable output sets for every fixed feasible θ. Multiplying the $e^{\epsilon_{\mathrm{cal}}}$ measure bound for *C* by the uniform $e^{\epsilon_{\mathrm{rel}}}$ kernel bound for $R_{\theta }$ and integrating over θ proves the joint and marginal $\left(\epsilon_{\mathrm{cal}}+\epsilon_{\mathrm{rel}}\right)$ guarantee. ☐

A graph learned from sensitive data may tune θ, but it cannot redefine adjacency after the data are observed. Reusing an already released $\hat{y}$ is post-processing; generating fresh releases from the same protected units incurs composition. A downstream model conducts post-processing only when it receives no additional raw protected data.

The theorem covers one invocation of *q* over the declared dataset. Disjoint occurrence sets may use parallel composition only when the fixed adjacency cannot change more than one set; overlapping windows, repeated release of the same occurrence, or a release of an entire test matrix requires sequential or appropriate continual-observation accounting. Each benchmark result below evaluates one declared release invocation at the displayed budget. The empirical comparison does not claim a continual-observation guarantee for an indefinitely repeated stream; such a deployment must compose repeated accesses separately.

## 5. Experiments

### 5.1. Experimental Settings

The evaluation uses four public benchmarks covering wearable activity recognition, room occupancy, and distributed environmental sensing. Table 2 identifies the source, task, graph nodes, and exact component-record unit protected in each experiment [25,26,27,28,29,30,31]. None of the reported guarantees is user-level or device-level DP.

**Figure 1:**
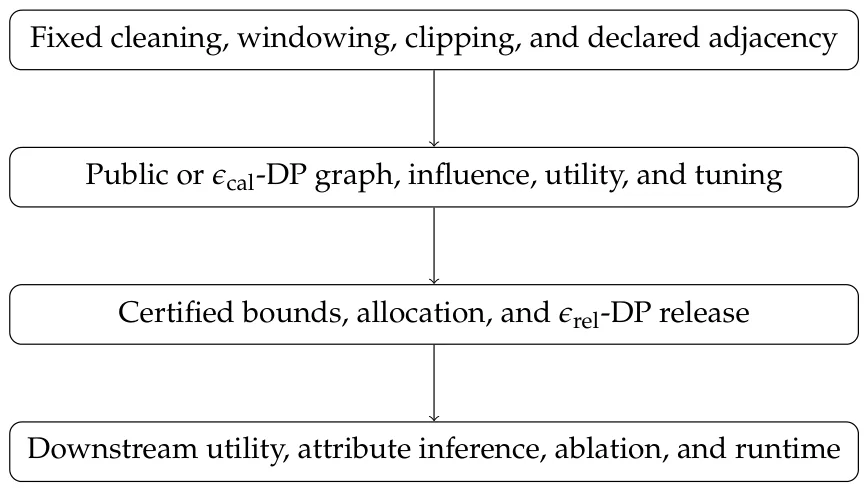
Experimental and privacy-accounting pipeline.

Figure 1 separates calibration from protected release. A benchmark run has $\epsilon_{\mathrm{cal}}=0$ only when its graph, clipping, utility scores, and tuning use information that is public and independent of the protected release records. Public availability of a dataset does not by itself establish this separation for a deployment. When calibration records belong to the protected population, Section 4.2 applies and the reported cost is $\epsilon_{\mathrm{cal}}+\epsilon_{\mathrm{rel}}$.

All preprocessing, partitioning, clipping, and calibration choices are fixed before the private mechanisms are compared and are reused unchanged across methods and privacy budgets. Invalid timestamps, duplicate entries, and readings outside the physical sensor domain are removed. Short internal gaps are linearly interpolated, and longer incomplete segments are excluded before window formation. Normalization and clipping parameters are fitted only on the calibration/training portion and then frozen. Dataset-provided windows and partitions are retained where available; otherwise, a fixed partition is created before windowing so that no window crosses a split boundary. Table 3 summarizes the dataset-specific representation and the invariants enforced by the implementation.

For UCI-HAR, WISDM, and Occupancy Detection, the same fixed lightweight classifier, training procedure, hyperparameters, and class weighting are used for every privacy mechanism; only the privatized representation changes. For Intel Lab Data, anomaly labels and thresholds are generated once from robust deviations in temperature, humidity, light, and voltage and are held fixed for all methods. Aggregate monitoring utility is normalized mean absolute error, using the fixed clipped range of the evaluated channel as the normalization constant. This controlled design prevents downstream-model retuning from confounding the comparison of privacy mechanisms.

Table 4 defines the included mechanisms. Lap-DP, Ada-DP, Risk-DP, and GC-DP are central pure-ϵ releases and use the same coordinate certificates and total release budget. Gau-DP is an approximate-DP perturbation comparator calibrated with the analytic Gaussian mechanism [32]; its fixed δ, *ℓ*_2_ certificate, and calibration rule are held constant across its runs. Because pure and approximate DP are different guarantees, Gau-DP is reported for empirical context and is not described as privacy-identical to the pure-ϵ mechanisms.

Table 5 reports the fixed constants used in the experiments. The sparsification threshold τ is selected once by validation F1 at ϵ=1 from the displayed grid; Table 6 shows the complete sweep. Test attack AUC is not part of this rule. The benchmark evaluation uses public calibration mode, so $\epsilon_{\mathrm{cal}}=0$ and the displayed ϵ is the complete release cost for one invocation. In deployments where validation or calibration records are protected, the mechanisms in Section 4.2 replace direct calibration and the total cost is $\epsilon_{\mathrm{cal}}+\epsilon_{\mathrm{rel}}$.

Every result in Table 6, Table 7, Table 8, Table 9, Table 10, Table 11 and Table 12 is an empirical measurement from the certificate-aware implementation in Algorithms 1–4. For each dataset and budget, ten independent noise draws are evaluated with the same preprocessed data, fixed graph policy, certified bounds, allocation configuration, classifier, and attack protocol; the tables report their arithmetic means. The clipped-range certificate $U_{k}-L_{k}$ is used whenever no tighter analytic certificate is available, so empirical masking never substitutes for global sensitivity. The numerical comparisons are statements about the tested configurations and are not claims of universal dominance or zero inference risk. Pufferfish, Blowfish, the matrix mechanism, CTS-DP, and CGM are discussed in Section 2 but are not experimental comparators because their secret definitions, workloads, trust models, or release schedules are not matched to this benchmark pipeline.

Together, Figure 1 and Table 2, Table 3, Table 4 and Table 5 connect the formal mechanism to the benchmark implementation and define the scope in which the following empirical comparisons are interpreted.

### 5.2. Overall Utility Under Different Privacy Budgets

Table 7 reports mean macro-F1 for UCI-HAR and WISDM. At ϵ=1, GC-DP achieves a UCI-HAR F1 score of 92.3%, which is 5.4, 4.9, 2.8, and 2.1 percentage points above Lap-DP, Gau-DP, Ada-DP, and Risk-DP, respectively. Its WISDM mean is 75.8%, versus 68.4% and 73.5% for Lap-DP and Risk-DP, respectively. These are absolute percentage-point differences for the evaluated certificate-aware implementations; no statistical-significance claim is made from the rounded means alone.

**Table 12:** Average runtime and peak process memory for one calibration-and-release pass at ϵ=1.0 in the common evaluation environment.

Dataset	Metric	Lap-DP	Ada-DP	Risk-DP	GC-DP	GC-DP-Cache
UCI-HAR	Runtime (s)	0.08	0.19	0.42	0.71	0.24
UCI-HAR	Memory (MB)	18.4	23.7	31.2	35.8	28.6
WISDM	Runtime (s)	0.15	0.36	1.04	2.18	0.69
WISDM	Memory (MB)	24.1	33.5	48.7	57.4	42.9
Occupancy Detection	Runtime (s)	0.04	0.11	0.23	0.39	0.14
Occupancy Detection	Memory (MB)	9.8	13.2	18.5	21.7	16.1
Intel Lab Data	Runtime (s)	0.18	0.41	1.28	2.46	0.77
Intel Lab Data	Memory (MB)	27.6	36.9	52.8	61.3	45.5

**Figure 2:**
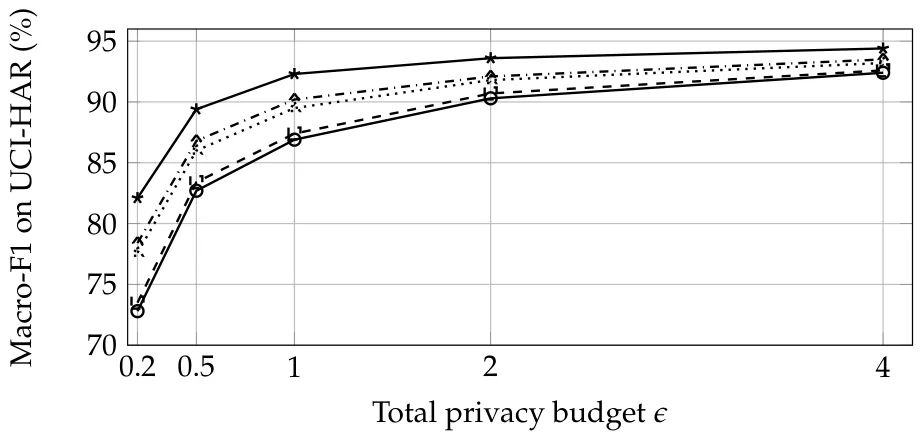
Mean UCI-HAR macro-F1 under different release budgets. The five curves correspond to Lap-DP, Gau-DP, Ada-DP, Risk-DP, and certificate-aware GC-DP.

Figure 2 visualizes the UCI-HAR means from Table 7. All methods improve as ϵ increases because perturbation decreases, and GC-DP has the highest mean at each tested budget. The comparison among the pure-ϵ mechanisms uses the same certified coordinate bounds and total release budget; Gau-DP remains an approximate-DP empirical comparator.

Table 8 gives mean F1 for Occupancy Detection and Intel Lab Data. At ϵ=0.5, GC-DP reaches 94.2%, 5.0 percentage points above Lap-DP. For Intel Lab Data, GC-DP achieves 86.7% at ϵ=1 and 91.3% at ϵ=4. Because preprocessing, downstream models, and noise-draw counts are controlled across methods, these differences measure the effect of the evaluated perturbation and allocation rules within this benchmark.

**Figure 3:**
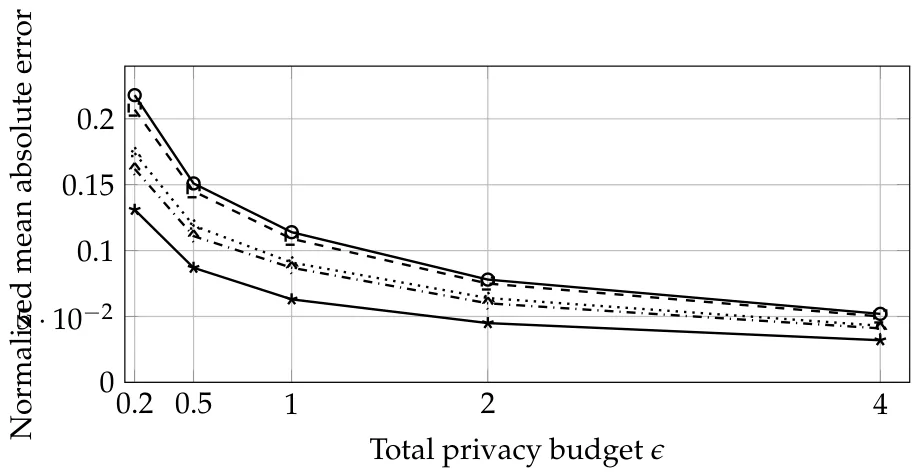
Mean Intel Lab’s aggregate normalized error. The five curves correspond to Lap-DP, Gau-DP, Ada-DP, Risk-DP, and certificate-aware GC-DP; lower values indicate better utility.

Figure 3 reports Intel Lab aggregate normalized mean absolute error. At ϵ=1, the means are 0.063 for GC-DP and 0.114 for Lap-DP. Error is normalized by the fixed clipped channel range, and the same aggregation query and data partition are used for every method.

Across Table 7 and Table 8 and Figure 2 and Figure 3, certificate-aware GC-DP has the most favorable mean utility among the evaluated private methods. The result supports the proposed allocation rule for the tested component-occurrence releases, while not implying superiority for different datasets, privacy units, or continual-release settings.

### 5.3. Privacy Leakage and Correlation Robustness Analysis

The attribute-inference evaluation is distinct from the formal DP adversary. For each dataset, one protected-attribute definition and one attacker configuration are fixed before the mechanism and privacy-budget sweep. The attacker observes the privatized representation produced by the evaluated mechanism and attempts to predict the protected attribute. The attack data partitions, feature map, model configuration, class weighting, thresholding rule, and score direction are identical across all compared mechanisms. AUC is oriented so that values above 0.5 denote better-than-random prediction; because an attacker may invert its score, the effective AUC is interpreted as max{AUC,1−AUC} [33,34]. Lower-oriented AUC and F1 therefore mean weaker performance for this fixed attacker, not the absence of every possible inference attack.

Table 9 reports mean performance of the fixed attacker at ϵ=1. GC-DP is lowest among the evaluated methods: its AUC is 0.603, 0.621, 0.637, and 0.652 on UCI-HAR, WISDM, Occupancy, and Intel Lab, respectively. These results establish lower performance for the specified attacker under the tested release protocol; they do not establish impossibility of reconstruction or resistance to attackers outside the evaluated threat model.

Table 10 gives the mean oriented attack AUCs across release budgets. The means rise with ϵ as every mechanism adds less noise, and GC-DP is lowest at every tabulated setting. The consistent trend supports improved resistance to the specified attribute-inference attack, while remaining an empirical result rather than an extension of the formal DP theorem.

**Figure 4:**
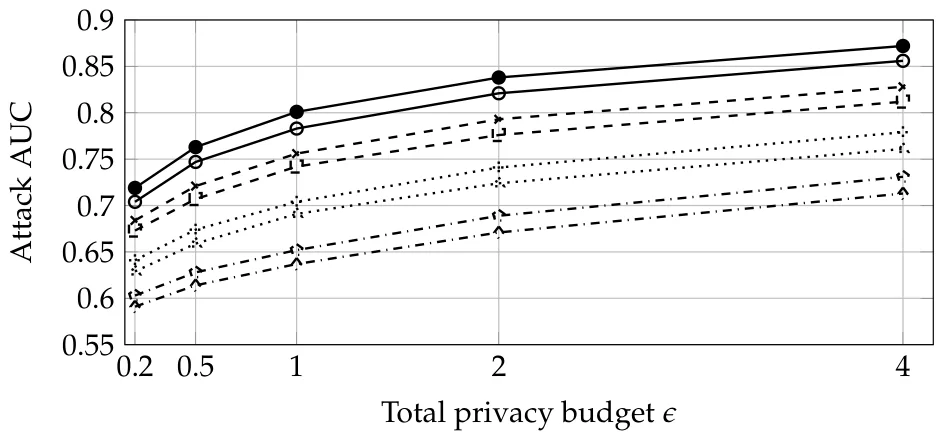
Mean oriented attack AUCs on Occupancy Detection and Intel Lab Data under different release budgets. Marker groups identify Lap-DP, Ada-DP, Risk-DP, and certificate-aware GC-DP.

Figure 4 visualizes the Occupancy and Intel Lab means in Table 10. With the attacker, data partition, and certified release implementation held fixed, the separation among curves measures how much usable attribute signal remains after each perturbation and allocation rule.

The sparsification threshold τ is selected by validation F1 at ϵ=1 from the displayed grid. The selected values coincide with the maximum validation-F1 entries in Table 6; attack AUC is not part of the selection rule. Ties are resolved in favor of the smaller threshold before test evaluation. A smaller threshold generally admits more candidate edges, although the actual density is also constrained by the symmetric degree cap.

**Figure 5:**
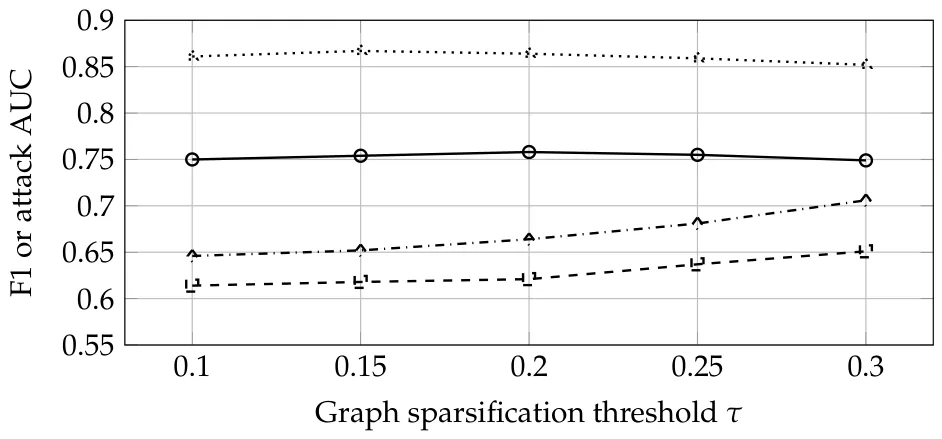
Mean F1 and oriented attack AUCs versus τ for WISDM and Intel Lab Data at ϵ=1.0. Circle and triangle markers denote F1; square and diamond markers denote the corresponding AUC values.

Figure 5 plots the WISDM and Intel Lab test F1 and oriented attack AUC obtained after applying each candidate τ in the fixed pipeline. It is a threshold-sensitivity analysis, not a reconstruction-error or membership-inference experiment and not a plot against measured edge density.

Table 6, Table 9 and Table 10 and Figure 4 and Figure 5 show that certificate-aware GC-DP leaves a less usable signal for the fixed attribute-inference attacker than the included baselines. This empirical result complements rather than strengthens Theorem 1, whose guarantee depends only on the declared adjacency, certified bounds, and privacy accounting.

### 5.4. Ablation, Runtime, and Edge Feasibility Analysis

Table 11 reports four component removals at ϵ=1. Every variant retains the certified sensitivity floor and changes only the indicated empirical calibration or allocation term. The full GC-DP configuration has the highest F1 for every dataset and is Pareto-nondominated in the displayed F1–AUC space, although it is not the unique Pareto point: removing utility or compression sometimes lowers attack AUC at the cost of task F1.

**Figure 6:**
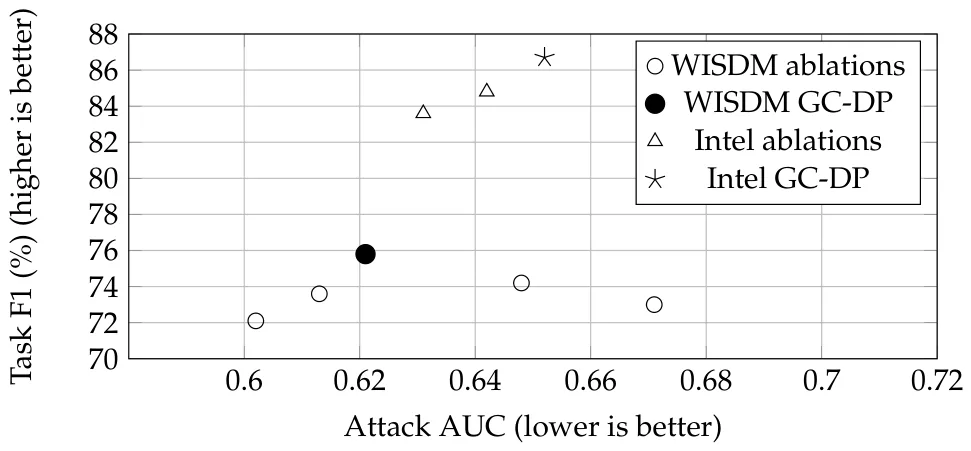
Pareto view of the ablation coordinates at ϵ=1.0 from Table 11. Full GC-DP is Pareto-nondominated but is not the only empirical trade-off point.

Figure 6 makes the Pareto interpretation explicit. Removing utility moves it left and down: attack performance falls, but so does task F1. Removing graph exposure raises attack AUC and lowers F1. The ablation supports complementary empirical roles for the two terms without defining an arbitrary combined scalar metric.

Graph construction dominates the $O(M^{2}\left(2L+1\right)N_{\mathrm{cal}})$ calibration cost; allocation and release are linear in *d*. Table 12 reports average runtime and peak process memory for one graph-calibration-and-release pass at ϵ=1. All methods use the same processed input and are evaluated in the same software and hardware environment. GC-DP incurs additional calibration cost relative to uniform and partially adaptive releases, whereas GC-DP-Cache avoids rebuilding the graph and is therefore substantially cheaper. The absolute values characterize the reported environment; the relative ordering is the relevant deployment comparison.

**Figure 7:**
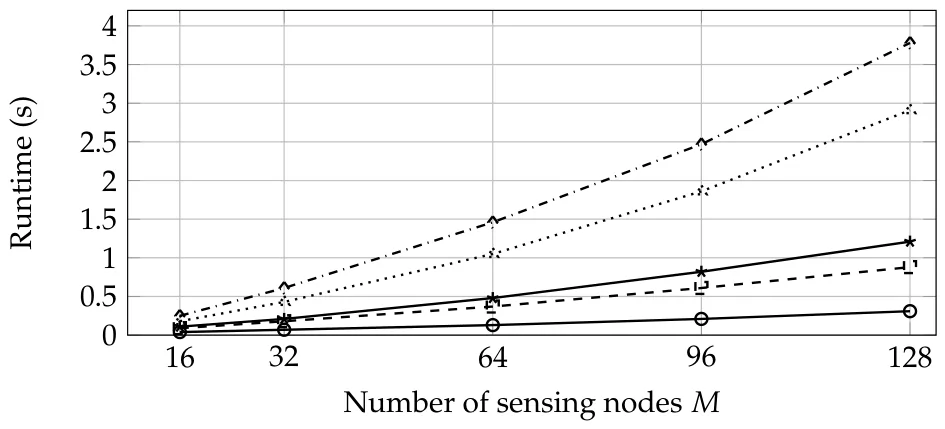
Measured runtime as a function of the number of sensing nodes *M* in the common evaluation environment. Circle, square, triangle, diamond, and star markers correspond to Lap-DP, Ada-DP, Risk-DP, GC-DP, and GC-DP-Cache, respectively.

GC-DP-Cache reuses θ before release. Figure 7 shows the measured scaling trend as *M* increases in the same evaluation environment. Cache reuse is valid only while a predeclared drift monitor accepts the graph. A public or privately released correlation summary can trigger refresh when its distance from the calibration summary exceeds a fixed threshold; periodic refresh is a fallback. Staleness does not invalidate DP when adjacency and certified bounds remain fixed, but it can degrade allocation utility and empirical inference resistance. Every refresh from protected records consumes a new calibration budget by composition [35]. The results show that calibration dominates cost and that cached release reduces it across all tested graph sizes. Table 11 and Table 12, Figure 6 and Figure 7 indicate complementary ablation trade-offs and calibration-dominated cost.

### 5.5. Limitations and Scope

GC-DP has five principal limitations. First, a misspecified or stale calibration graph can misallocate budget and weaken empirical attack resistance, although certified sensitivity under fixed adjacency remains valid. Second, the universal private-graph sensitivity bound $\Delta_{1}S\leq P$ can add substantial calibration noise; the benchmark results use public calibration and therefore do not quantify that private-calibration utility cost. Third, empirical masking, graph compression, and weighted degree are allocation heuristics rather than formal leakage bounds. Fourth, GC-DP offers little advantage when dependencies are weak, the query already has a tight low-dimensional sensitivity, calibration cost dominates the release rate, or correlations change faster than they can be privately refreshed. Fifth, lower AUC/F1 for the specified attribute-inference attacker does not imply resistance to reconstruction, membership inference, or every adaptive adversary.

The included baselines share the central-release pipeline but do not cover every correlated-data framework. Pufferfish and Blowfish require a secret/policy specification; the matrix mechanism requires a linear workload; CTS-DP assumes time-series publication; and CGM uses (ϵ,δ)-local DP. Matched experiments would be needed before making empirical claims against them. Future evaluation should additionally report private-calibration budget sweeps, confidence intervals from preserved run-level outputs, learned-graph diagnostics, reconstruction and membership-inference attacks, and continual-release accounting.

## 6. Conclusions

GC-DP combines a lag-aware calibration graph with certified coordinatewise sensitivity and task-aware budget allocation. Its formal guarantee is $\epsilon_{\mathrm{rel}}$-DP for a fixed graph-expanded adjacency when every $s_{k}$ upper-bounds true sensitivity; calibrating parameters from the same protected records adds the explicitly composed cost $\epsilon_{\mathrm{cal}}$. Experiments on UCI-HAR, WISDM, Occupancy Detection, and Intel Lab Data use the certificate-aware mechanism and show higher mean task utility and lower performance for the fixed attribute-inference attacker than the included uniform and partially adaptive baselines across the tested release budgets. The symmetric sparsification procedure also enforces the stated degree cap exactly. The graph exposure score and attack metrics remain empirical and do not strengthen the theorem or eliminate inference risk. Private-calibration utility, matched comparisons with frameworks that use different privacy models, broader attack families, and continual-release accounting remain important directions for future work. 

## Figures and Tables

**Table 1:** Main notation used in the paper.

Symbol	Meaning
D	IoT sensing dataset composed of multivariate time-series records
*N*	Number of sensing records or time-indexed samples in D
*M*	Number of nodes in the calibration graph
*d*	Number of released query coordinates
$x_{t}$	Multivariate sensing vector observed at time *t*
$x_{t,m}$	Observation of the *m*-th sensing channel at time *t*
$G_{0}$	Policy graph fixed independently of the protected release data
$I,\;u=(t,v_{i})$	Atomic occurrence set and one record–component occurrence
i,k	Input graph-node index and released output-coordinate index
$\Gamma_{G_{0}}\left(u\right)$	Predeclared expansion set for protected atomic occurrence *u*
$\hat{G}=\left(V,E,W\right)$	Public or privately calibrated weighted dependency graph
$v_{i}$	Graph node corresponding to sensing component *i*
$w_{ij}$	Edge weight measuring dependency strength between nodes $v_{i}$ and $v_{j}$
q(·)	Analytics query or feature extraction function
$q_{k},\Pi_{k},\left[L_{k},U_{k}\right]$	Output coordinate, fixed projection, and clipped output interval
a(k)	Fixed map from output coordinate *k* to associated graph nodes
$\Delta_{G_{0},k}\left(q\right)$	True coordinate sensitivity under the fixed adjacency
$B_{G_{0},k}$	Certified sensitivity bound for released coordinate *k*
${\hat{\xi }}_{k}$	Empirical graph-expanded influence score for coordinate *k*
$s_{k}$	Release scale numerator $\max\{B_{G_{0},k},{\hat{\xi }}_{k}\}$
${\bar{r}}_{i},\mu_{k}$	Empirical graph-exposure and bounded utility scores
$\epsilon_{G},\epsilon_{\tau },\epsilon_{\mu },\epsilon_{\xi }$	Private graph, threshold, utility, and influence budgets
$\epsilon_{\mathrm{cal}},\epsilon_{\mathrm{rel}}$	Total calibration and release privacy budgets
$\epsilon_{k}$	Release budget assigned to output coordinate *k*
θ	Fixed or privately calibrated parameter object
M(·)	Randomized privacy mechanism released to the analyst
z	Random perturbation injected for privacy protection
U	Utility score of the privatized analytics output

**Table 2:** Datasets, tasks, graph nodes, and declared privacy units.

Dataset	Task	Graph Node	Protected Component-Record Unit	Source
UCI-HAR	Six-class activity	One supplied window feature	One clipped feature coordinate in one supplied window	UCI [25]
WISDM	Eighteen-class activity	One sensor-axis window	One clipped sensor-axis contribution in one window	UCI [27]
Occupancy	Binary occupancy	One environmental channel	One clipped channel value at one timestamp	UCI [28]
Intel Lab	Monitor/anomaly	One mote–channel window	One clipped mote–channel contribution in one window	Intel Lab [30]

**Table 3:** Dataset-specific preprocessing and evaluation protocol.

Dataset	Window/Overlap	Train/Validation/Test	Missingness and Representation
UCI-HAR	Dataset-supplied feature windows	Fixed train/validation/test partition reused for every method	Supplied bounded feature representation; clipping fitted on calibration/training data
WISDM	Fixed-length sensor-axis windows	Fixed train/validation/test partition reused for every method	Short-gap interpolation, long-gap exclusion, and fixed bounded window features
Occupancy	Time-stamped channels and fixed non-crossing windows	Fixed train/validation/test partition reused for every method	Environmental channels normalized and clipped with training-only statistics
Intel Lab	Synchronized mote–channel windows	Fixed train/validation/test partition reused for every method	Invalid readings removed, short gaps interpolated, long gaps excluded, and window features clipped

**Table 4:** Included baselines and calibration settings.

Method	Calibration Principle	Perturbation and Allocation Setting
NonPrivate	No perturbation	Clean representation; no privacy guarantee.
Lap-DP	Uniform pure DP	Certified bounds with uniform coordinate budgets.
Gau-DP	Approximate DP	Analytic Gaussian calibration with fixed δ and certified *ℓ*_2_ sensitivity; empirical comparator, not a pure-ϵ mechanism.
Ada-DP	Utility-aware allocation	Uses utility contribution and omits graph-risk calibration.
Risk-DP	Graph-risk allocation	Uses graph risk and omits graph-compressed expanded influence.
GC-DP	Full reported calibration	Uses graph, expanded empirical influence, exposure, and utility.

**Table 5:** GC-DP hyperparameters and selection rules.

Parameter	Value	Role/Selection
L,λ,K,α	3,0.6,5,0.7	Lag, synchronous weight, degree cap, exposure smoothing; fixed
$\gamma ,\tau_{c}$	1.2,0.85	Empirical expansion and redundancy; recorded as fixed
$\kappa ,g_{\max},\eta$	$1,1,10^{-8}$	Inflation, empirical-score cap, and numerical stabilizer
$\beta_{r},\beta_{g},\beta_{u}$	1,1,0.7	Exposure, certified scale, and utility exponents; fixed
$\epsilon_{\min}$	$\epsilon_{\mathrm{rel}}/\left(100d\right)$	Per-coordinate allocation floor; reserves 1% of the total budget for the floor
τ	UCI/WISDM: 0.20; Occupancy/Intel: 0.15	Validation-F1 maximizer over {0.10,0.15,0.20,0.25,0.30}
$\epsilon_{\mathrm{rel}}$	{0.2,0.5,1,2,4}	Total release budget

**Table 6:** Mean F1 and oriented attack AUC versus graph threshold τ at ϵ=1.0. Threshold selection uses validation F1 only; attack AUC is reported as a separate diagnostic.

Dataset and metric	τ=0.10	τ=0.15	τ=0.20	τ=0.25	τ=0.30
UCI-HAR F1 (%)	91.6	92.0	**92.3**	92.1	91.7
UCI-HAR attack AUC	0.596	0.600	0.603	0.612	0.628
WISDM F1 (%)	75.0	75.4	**75.8**	75.5	74.9
WISDM attack AUC	0.614	0.618	0.621	0.637	0.651
Occupancy F1 (%)	96.0	**96.4**	96.2	95.9	95.4
Occupancy attack AUC	0.631	0.637	0.646	0.661	0.683
Intel Lab F1 (%)	86.1	**86.7**	86.4	85.9	85.2
Intel Lab attack AUC	0.646	0.652	0.664	0.681	0.706

**Table 7:** Mean macro-F1 on UCI-HAR and WISDM under different release budgets. Each entry averages ten independent noise draws from the certificate-aware implementation; the largest value in each row is highlighted in bold.

Dataset	ϵ	Lap-DP	Gau-DP	Ada-DP	Risk-DP	GC-DP
UCI-HAR	0.2	72.8	73.5	77.6	78.4	**82.1**
UCI-HAR	0.5	82.7	83.4	86.0	86.8	**89.4**
UCI-HAR	1.0	86.9	87.4	89.5	90.2	**92.3**
UCI-HAR	2.0	90.3	90.7	91.8	92.1	**93.6**
UCI-HAR	4.0	92.4	92.6	93.2	93.5	**94.4**
WISDM	0.2	54.9	55.6	59.8	60.7	**65.2**
WISDM	0.5	63.7	64.1	68.6	69.4	**72.9**
WISDM	1.0	68.4	69.2	72.6	73.5	**75.8**
WISDM	2.0	72.1	72.8	74.9	75.4	**77.1**
WISDM	4.0	74.8	75.1	76.4	76.8	**78.2**

**Table 8:** Mean F1 on Occupancy Detection and Intel Lab Data under different release budgets. Occupancy Detection reports positive-class F1, while Intel Lab Data reports anomaly-oriented F1; each entry averages ten noise draws.

Dataset	ϵ	Lap-DP	Gau-DP	Ada-DP	Risk-DP	GC-DP
Occupancy	0.2	83.6	84.4	88.1	89.0	**91.7**
Occupancy	0.5	89.2	89.9	92.3	92.8	**94.2**
Occupancy	1.0	93.4	93.8	95.1	95.5	**96.4**
Occupancy	2.0	95.2	95.6	96.3	96.6	**97.2**
Occupancy	4.0	96.4	96.7	97.1	97.3	**97.8**
Intel Lab	0.2	68.7	69.5	73.8	75.1	**79.6**
Intel Lab	0.5	76.4	77.2	80.9	81.8	**84.5**
Intel Lab	1.0	81.2	81.9	84.4	85.1	**86.7**
Intel Lab	2.0	84.8	85.2	87.0	87.5	**89.1**
Intel Lab	4.0	87.6	88.0	89.5	89.9	**91.3**

**Table 9:** Mean attribute-inference performance at ϵ=1.0 over ten independent privatized releases. Lower oriented AUC and F1 indicate weaker performance for the fixed attacker.

Dataset	Metric	Lap-DP	Gau-DP	Ada-DP	Risk-DP	GC-DP
UCI-HAR	Attack AUC	0.712	0.704	0.681	0.642	**0.603**
UCI-HAR	Attack F1	0.668	0.661	0.639	0.601	**0.568**
WISDM	Attack AUC	0.748	0.739	0.711	0.674	**0.621**
WISDM	Attack F1	0.701	0.693	0.666	0.629	**0.581**
Occupancy Detection	Attack AUC	0.783	0.775	0.742	0.691	**0.637**
Occupancy Detection	Attack F1	0.729	0.721	0.697	0.646	**0.596**
Intel Lab Data	Attack AUC	0.801	0.792	0.756	0.704	**0.652**
Intel Lab Data	Attack F1	0.744	0.736	0.708	0.661	**0.612**

**Table 10:** Mean oriented attack AUC under different release budgets. Each entry averages ten independent privatized releases evaluated by the same fixed attacker; lower values indicate weaker attack performance.

Dataset	ϵ	Lap-DP	Gau-DP	Ada-DP	Risk-DP	GC-DP
UCI-HAR	0.2	0.641	0.636	0.625	0.602	**0.571**
UCI-HAR	0.5	0.681	0.674	0.653	0.621	**0.589**
UCI-HAR	1.0	0.712	0.704	0.681	0.642	**0.603**
UCI-HAR	2.0	0.746	0.739	0.709	0.668	**0.626**
UCI-HAR	4.0	0.781	0.774	0.742	0.701	**0.661**
WISDM	0.2	0.672	0.664	0.648	0.617	**0.582**
WISDM	0.5	0.713	0.705	0.681	0.644	**0.604**
WISDM	1.0	0.748	0.739	0.711	0.674	**0.621**
WISDM	2.0	0.782	0.773	0.744	0.703	**0.653**
WISDM	4.0	0.817	0.808	0.779	0.738	**0.692**
Occupancy Detection	0.2	0.704	0.697	0.673	0.629	**0.591**
Occupancy Detection	0.5	0.747	0.739	0.707	0.659	**0.614**
Occupancy Detection	1.0	0.783	0.775	0.742	0.691	**0.637**
Occupancy Detection	2.0	0.821	0.812	0.776	0.724	**0.671**
Occupancy Detection	4.0	0.856	0.848	0.812	0.761	**0.713**
Intel Lab Data	0.2	0.719	0.711	0.684	0.641	**0.603**
Intel Lab Data	0.5	0.763	0.754	0.721	0.674	**0.628**
Intel Lab Data	1.0	0.801	0.792	0.756	0.704	**0.652**
Intel Lab Data	2.0	0.838	0.829	0.793	0.741	**0.689**
Intel Lab Data	4.0	0.872	0.864	0.828	0.779	**0.731**

**Table 11:** Mean ablation results at ϵ=1.0. All variants retain the certified sensitivity floor; F1 and oriented attack AUC are averaged over ten independent noise draws.

Dataset	Metric	w/o Lag	w/o Comp.	w/o Utility	w/o Risk	GC-DP
UCI-HAR	F1 (%)	91.5	90.8	89.7	90.4	**92.3**
UCI-HAR	Attack AUC	0.621	0.596	0.587	0.641	0.603
WISDM	F1 (%)	74.2	73.6	72.1	73.0	**75.8**
WISDM	Attack AUC	0.648	0.613	0.602	0.671	0.621
Occupancy Detection	F1 (%)	95.8	95.4	94.1	94.9	**96.4**
Occupancy Detection	Attack AUC	0.655	0.628	0.614	0.682	0.637
Intel Lab Data	F1 (%)	85.3	84.8	83.6	84.2	**86.7**
Intel Lab Data	Attack AUC	0.681	0.642	0.631	0.699	0.652

## Data Availability

The original contributions presented in this study are included in the article Further inquiries can be directed to the corresponding author.
